# Distribution of fluoroquinolone resistance determinants in Carbapenem-resistant *Klebsiella pneumoniae* clinical isolates associated with bloodstream infections in China

**DOI:** 10.1186/s12866-021-02238-7

**Published:** 2021-06-02

**Authors:** Qing Zhan, Yanlei Xu, Bingjie Wang, Jingyi Yu, Xiaofei Shen, Li Liu, Xingwei Cao, Yinjuan Guo, Fangyou Yu

**Affiliations:** 1 Jiangxi Provincial Key Laboratory of Preventive Medicine, School of Public Health, Nanchang University, Nanchang, 330006 P. R. China; 2 Department of Clinical Laboratory Medicine, Shanghai Pulmonary Hospital, Tongji University School of Medicine, Shanghai, 200082 P. R. China; 3 Department of Laboratory Medicine, the First Affiliated Hospital of Wenzhou Medical University, Wenzhou, 325000 P. R. China; 4 Department of Respiratory Medicine, the First Affiliated Hospital of Wenzhou Medical University, Wenzhou, 325000 P. R. China; 5 Shanghai Key Laboratory of Tuberculosis, Shanghai Pulmonary Hospital, Tongji University School of Medicine, Shanghai, 200082 P. R. China

**Keywords:** CRKP, Bloodstream infections, Fluoroquinolones, PMQR, QRDR, ST11

## Abstract

**Background:**

The rate of fluoroquinolone (FQ) resistance among carbapenem-resistant *Klebsiella pneumoniae* (CRKP) is high. The present study aimed to investigate the distribution of fluoroquinolone resistance determinants in clinical CRKP isolates associated with bloodstream infections (BSIs).

**Results:**

A total of 149 BSI-associated clinical CRKP isolates collected from 11 Chinese teaching hospitals from 2015 to 2018 were investigated for the prevalence of fluoroquinolone resistance determinants, including plasmid-mediated quinolone resistance (PMQR) genes and spontaneous mutations in the quinolone resistance-determining regions (QRDRs) of the *gyrA* and *parC* genes. Among these 149 clinical CRKP isolates, 117 (78.5%) exhibited resistance to ciprofloxacin. The GyrA substitutions (Ser83 → IIe/Phe) and (Asp87 → Gly/Ala) were found among 112 (75.2%) of 149 isolates, while the substitution (Ser80 → IIe) of ParC was found in 111 (74.5%) of the 149 isolates. In total, 70.5% (105/149) of the CRKP isolates had at least two mutations within *gyrA* as well as a third mutation in *parC*. No mutations in the QRDRs were found in 31 ciprofloxacin susceptible CRKP isolates. Eighty-nine (56.9%) of 149 were found to carry PMQR genes including *qnrS1* (43.0%), *aac(6′)-Ib-cr* (16.1%), *qnrB4* (6.0%), *qnrB2* (2.7%), and *qnrB1* (1.3%). Nine isolates contained two or more PMQR genes, with one carrying four [*aac(6′)-Ib-cr*, *qnr-S1*, *qnrB2,* and *qnrB4*]. The co-existence rate of PMQR determinants and mutations in the QRDRs of *gyrA* and *parC* reached 68.5% (61/89). Seventy-four (83.1%, 74/89) PMQR-positive isolates harbored extended-spectrum beta-lactamase (ESBL)-encoding genes. Multilocus sequence typing (MLST) analysis demonstrated that the ST11 was the most prevalent STs in our study.

**Conclusions:**

Mutations in the QRDRs of *gyrA* and *parC* were the key factors leading to the high prevalence of fluoroquinolone resistance among BSI-associated CRKP. The co-existence of PMQR genes and mutations in the QRDRs can increase the resistance level of CRKP to fluoroquinolones in clinical settings. ST11 CRKP isolates with identical QRDR substitution patterns were found throughout hospitals in China.

## Introduction

*Klebsiella pneumoniae* is a commonly detected pathogen in hospital settings, causing nosocomial and community-acquired infections in the lung, urinary tract, surgical sites, soft tissue infections and the bloodstream [1]. CRKP has emerged as a worldwide problem, posing major challenges for its clinical management and public health, through its ability to cause severe and untreatable infections in otherwise healthy individuals [2, 3]. In particular, BSIs caused by CRKP is associated with high mortality due to the ineffectiveness of antibacterials used to treat them [4]. CRKP usually shows high levels of resistance to many types of antibiotics [5]. The optimal treatment options for CRKP infections are not well defined. They currently include the use of older agents either as monotherapy or in combination with drugs such as fluoroquinolones (FQs) [6, 7].

FQs are important synthetic antimicrobial agents extensively used in clinical and veterinary medicine. They exhibit broad-spectrum activity against a wide of important clinical pathogens and exhibit excellent tissue penetration [8–10]. To reduce the use of carbapenems, FQs have been proposed as first-choice alternatives in the treatment of FQ-susceptible, ESBL-producing enterobacterial organisms in pyelonephritis [11]. Carbapenems with FQ are also used to treat carbapenem non-susceptible *K. pneumoniae* infections [12, 13]. However, resistance to FQs has increased rapidly due to their overuse, thereby limiting the available treatment options or leading to treatment failure [14, 15]. FQs target DNA gyrase A and topoisomerase IV, which are encoded by *gyrA* and *parC*, respectively. The biological mechanisms of resistance to FQs include impermeability, active efflux, target modification, and antibiotic neutralization. Two major mechanisms involved in the development of quinolone resistance are the acquisition of plasmid-mediated quinolone resistance (PMQR) genes [such as *aac(6′)-Ib-cr* and *qnr*] and spontaneous mutations in quinolone resistance-determining regions (QRDRs) of *gyrA* and *parC* [16–18]. PMQR genes have recently been shown to confer low-level resistance to FQs and can be horizontally transferred [19]. The co-carriage of mutations in QRDRs and PMQR genes has been reported in clinical isolates of *Enterobacteriaceae* exhibiting high-level quinolone resistance [18, 20, 21]. Alterations in both *gyrA* and *parC* often confer high-level resistance and are reported more frequently than those in *gyrB* or *parE* [16]. However, relatively few studies have assessed the prevalence of PMQR determinants and the diversity of DNA gyrase and topoisomerase IV mutations in clinical isolates of CRKP associated with BSIs in China [14, 22]. Accordingly, the current study aimed to investigate the prevalence, molecular characteristics, and distribution of PMQR determinants and mutations in the QRDRs of *gyrA* and *parC* BSI-associated clinical CRKP isolates from 11 hospitals in China.

## Materials and methods

### Collection and identification of clinical *K. pneumoniae* isolates

From April 2015 to November 2018, a total of 149 CRKP isolates were cultured from the blood of patients with BSIs in 11 hospitals in eight provinces of China, including Zhejiang (*n* = 22), Fujian (*n* = 9), Shandong (*n* = 26), Hubei (*n* = 10), Henan (*n* = 16), Shanghai (*n* = 18), Jiangxi (*n* = 40), and Hunan (*n* = 8). These *K. pneumoniae* isolates were identified by Gram-staining and a VITEK-2 automated platform (bioMérieux, Marcy l’Etoile, France) according to the manufacturer’s instructions, as well as by additional biochemical testing. CRKP isolates were selected based on resistance to imipenem or meropenem according to Clinical and Laboratory Standards Institute (CLSI) guidelines [23]. *Escherichia coli* ATCC25922 and *Pseudomonas aeruginosa* ATCC27853 were used as control isolates for the identification and antimicrobial susceptibility testing of bacterial clinical isolates.

### Antimicrobial susceptibility testing

MICs of carbapenem (imipenem, meropenem), fluoroquinolones (ciprofloxacin), aminoglycosides (amikacin and gentamicin), β-lactams/β-lactamase inhibitor complexes (ceftazidime-avibactam and piperacillin-tazobactam), cephalosporins (ceftazidime, cefepime, cefotaxime, and cefoxitin), folate metabolic pathway inhibitors (sulfamethoxazole), polymyxin B, tetracyclines (tigecycline, minocycline, and tetracycline), and monocyclic β-lactam (aztreonam) were determined by the broth microdilution method, according to Clinical and Laboratory Standards Institute (CLSI) guidelines [23]. The results were interpreted according to CLSI breakpoints. *E. coli* ATCC 25922 was used as a control isolate for antimicrobial susceptibility testing.

### PCR detection and DNA sequence analyses of QRDR and PMQR

Genomic DNA (gDNA) of the 149 CRKP isolates was extracted using the Ezup Column Bacteria Genomic DNA Purification Kits (Sangon Biotech, Shanghai, China) according to the manufacturer’s instructions. The Qubit and Nanodrop were used to determine the concentrations and purity of the extracted gDNA. The nucleotide mutations in the QRDRs of *gyrA* and *parC* were further tested by PCR and nucleotide sequencing using previously primers described [24]. Nucleotide mutations were identified based on the available nucleotide sequences of *gyrA* and *parC* genes of *K. pneumoniae* ATCC 13833. Sequence alignment and analysis were performed online using the BLAST program (http://www.ncbi.nlm.nih.gov). Plasmids were extracted from the 149 CRKP isolates using a Plasmid Midi Kit (Qiagen, Germany), and all the isolates were screened for the presence of the PMQR genes, including *qnrA, qnrB, qnrS, qepA,* and *aac(6′)-Ib-cr*, by PCR and DNA sequencing [25].

### The detection of carbapenem resistance genes and ESBLs-encoding genes

Carbapenemase genes (*bla*_*KPC*_, *bla*_*IMP*_, *bla*_*VIM*_*, bla*_*NDM*_, *bla*_*OXA-48*_) and ESBLs-producing genes (*bla*_*CTX-M*_*, bla*_*SHV*_, *bla*_*TEM*_) of all CRKP isolates were detected by PCR using gene-specific primers for each one, as previously reported [26, 27].

### Multilocus sequence typing

Multilocus sequence typing (MLST) was performed on all 149 CRKP isolates using primers targeting seven standard housekeeping genes (*gapA, infB, mdh, pgi, phoE, rpoB* and *tonB*) listed on the PubMLST website (http://www.pasteur.fr/recherche/genopole/PF8/mlst/Kpneumoniae.html) according to previously published methods, and the sequence types (STs) were determined using the MLST database [28].

### Statistical analysis

The data obtained for all CRKP isolates harboring different FQs resistance mechanisms were analyzed by SPSS software (version 20, IBM SPSS Statistics). The chi-square test was used for categorical variables. *P*-values < 0.05 were considered significant.

All the experiments were carried out following the relevant guidelines and regulations.

## Results

### The resistance of CRKP isolates to ciprofloxacin

Among 149 *K. pneumoniae* isolates, 117 (79.4%) exhibited resistance to ciprofloxacin, whereas only 31 (20.8%) showed susceptibility to ciprofloxacin (MICs of ≦1 μg/ml), and one showed intermediate resistance to ciprofloxacin (MIC of 2 μg/ml).

### The prevalence of mutations in the QRDRs of *gyrA* and *parC* among clinical CRKP isolates

Among the 149 CRKP isolates, the nucleotide mutations in QRDRs were detected in 112 (75.2%) isolates. Substitutions of QRDRs were observed at position 83 (102 isolates with Ser83 → IIe and 10 isolates with Ser83 → Phe) and position 87 (97 isolates with Asp87 → Gly and 10 isolates with Asp87 → Ala) of GyrA. The substitutions of Ser83 → Phe and Asp87 → Ala substitutions co-existed in 10 ciprofloxacin-resistant isolates. Mutations in *parC* were only found at position 80 (Ser80 → IIe) among 111 (74.5%) of the 149 isolates (Table 1). No *gyrB* or *parE* mutations were observed in any of the CRKP isolates.

**Table 1:** Patterns and distribution of GyrA and ParC substitutions and PMQR in 149 clinical CRKP isolates

CIP (MIC)	Mutation	Carrying PMQR (frequency)	Frequency	MLST (number of isolates)
S	No substitution	*aac(6′)-Ib-cr* (12); *qnrS1*(9); *qnrB1* (1); *aac(6′)-Ib-cr*, *qnrS1* (1); *aac(6′)-Ib-cr*, *qnrS1*, *qnrB4* (1)	31	ST107 (1); ST1319 (2); ST2390 (1); ST290 (7); ST307 (3); ST35 (2); ST37 (1); ST375 (1); ST45 (12); ST462 (1)
I	ParC-80I; GyrA-83I; GyrA-87G	*qnrS1* (1)	1	ST11 (1)
R	No substitution	*aac(6′)-Ib-cr* (1); *qnrS1*(1); *qnrB4* (1); *aac(6′)-Ib-cr*, *qnrS1* (1); *aac(6′)-Ib-cr*, *qnrS1, qnrB2* (1)	6	ST1692 (2); ST290 (1); ST290 (1); ST2236 (1)
R	ParC-80I; GyrA-83F; GyrA-87A	–	4	ST15
R	ParC-80I; GyrA-83F; GyrA-87A	*aac(6′)-Ib-cr* (2); *qnrB4* (2): *aac(6′)-Ib-cr*, *qnrS1*, *qnrB1* (1); *aac(6′)-Ib-cr*, *qnrB2* (1)	6	ST15 (4); ST2237 (2)
R	ParC-80I; GyrA-83I; GyrA-87G	–	47	ST11 (47)
R	ParC-80I; GyrA-83I; GyrA-87G	*qnrS1* (44); *qnrB4* (3); *aac(6′)-Ib-cr*, *qnrB1, qnrB4* (1)	48	ST11 (48)
R	GyrA-83I; GyrA-87G	*qnrS1* (1)	1	ST11
R	ParC-80I; GyrA-83I	*aac(6′)-Ib-cr* (1); *qnrS1* (2); *aac(6′)-Ib-cr*, *qnrS1*, *qnrB2*, *qnrB4* (1) *aac(6′)-Ib-cr*, *qnrB2* (1)	5	ST438 (2); ST485 (1); ST11 (1); ST395 (1)

*CIP* ciprofloxacin, *MIC* minimum inhibitory concentration, *R* resistance, *I* intermediate, *S* sensitive, *PMQR* plasmid-mediated quinolone resistance, *CRKP* carbapenem-resistant *Klebsiella pneumoniae*

There were 105 ciprofloxacin resistant CRKP isolates co-carried at least two mutations within *gyrA* and one in *parC*. And the 105 isolates with multiple mutations in QRDRs were distributed in 8 provinces, including Jiangxi (*n* = 38), Shandong (*n* = 10), Hubei (*n* = 7), Henan (*n* = 13), Shanghai (*n* = 16), Zhejiang (*n* = 9), Fujian (*n* = 9), and Hunan (*n =* 3).

### The prevalence of PMQR determinants among the 149 CRKP isolates

Among the 149 CRKP isolates tested, 89 (56.9%, 89/149) including 73.0% (65/89) of ciprofloxacin-resistant isolates were found to carry at least one PMQR gene, including *qnrS1* (71.9%, 64/89), *aac(6′)-Ib-cr* (27.0%, 24/89), *qnrB4* (10.1%, 9/89), *qnrB2* (4.5%, 4/89) and *qnrB1* (3.4%, 3/89). Nine isolates harbored two or more PMQR genes (Table 1). The *qnr* genes (59.7%, 74/149) were the major PMQR determinants and included 2 *qnr* families (*qnrB* and *qnrS)*. Twenty-eight (31.5%) of the 89 isolates with PMQR genes, 4 of which were resistant to ciprofloxacin, did not have mutations in the QRDRs. Among the 31 ciprofloxacin-susceptible isolates, 8 with no PMQR genes had ciprofloxacin MICs of ≤0.25 μg/ml, which were lower than those found for the 23 isolates carrying PMQR genes (Table 2).

**Table 2:** The role of PMQR in 31 FQ-sensitive CRKP isolates

Number of isolates	Carrying PMQR (frequency)	Mutation in QRDRs	CIP MIC (μg/mL)
8	–	No substitution	≤0.25 (8 isolates)
23	*aac(6′)-Ib-cr* (11)*;* *qnrS1* (9); *qnrB1* (1); *aac(6′)-Ib-cr*, *qnrS1* (11); *aac(6′)-Ib-cr*, *qnrS1*, *qnrB4* (1)	No substitution	0.5 (10 isolates) 1 (1 isolate) ≤0.25 (12 isolates)

*CIP* ciprofloxacin, *MIC* minimum inhibitory concentration, *PMQR* plasmid-mediated quinolone resistance, *CRKP* carbapenem-resistant *Klebsiella pneumoniae*, *FQ* fluoroquinolone

The 89 isolates carrying PMQR genes were distributed in 8 provinces, including Jiangxi (*n* = 29), Shandong (*n* = 13), Hubei (*n* = 3), Henan (*n* = 8), Shanghai (*n* = 10), Zhejiang (*n* = 16), Fujian (*n* = 8), and Hunan (*n* = 2). The 66 isolates harboring *qnr* family genes were mainly distributed in Jiangxi and Zhejiang and 14 with *aac(6′)-Ib-cr* were found mainly in Shandong.

### The co-existence of PMQR and mutations in QRDRs

The co-existence rate of PMQR determinants and mutations in the QRDRs of *gyrA* and *parC* were relatively high (68.5%, 61/89), with 60 being resistant to ciprofloxacin, and one showing intermediary resistance to ciprofloxacin. The patterns of co-carrying of PMQR and mutations in QRDRs could be found in Table 1, Mainly 45 (73.7%) carried qnrS1 and mutations in gyrA (Ser83 → IIe, Asp87 → Gly) and parC (Ser80 → IIe). These 61 isolates were distributed in 7 provinces, including Jiangxi (*n* = 29), Hubei (*n* = 3), Henan (*n* = 6), Shanghai (*n* = 8), Zhejiang (*n* = 6), Fujian (*n* = 8), and Hunan (*n* = 1).

### Molecular characteristics of the 149 clinical CRKP isolates

Six STs were identified among 112 CRKP isolates harboring mutations in the QRDRs of *gyrA* and *parC*. ST11 was the most prevalent ST (86.6%, 97/112), followed by ST15 (7.1%, 8/112), ST2237 (1.8%, 2/112), and ST438 (1.8%, 2/112). ST485 and ST395 were each found in only one isolate (Table 1).

Ninety-five ST11 CRKP isolates with identical mutations in the QRDRs were distributed in 8 provinces, including Jiangxi (*n* = 38), Shandong (*n* = 10), Hubei (*n* = 7), Henan (*n* = 10), Shanghai (*n* = 15), Zhejiang (*n* = 3), Fujian (*n* = 9), and Hunan (*n* = 3).

### Carbapenem resistance genes profiles, and antimicrobial resistance among PMQR-positive and PMQR-negative CRKP isolates

Among the 89 PMQR-positive CRKP isolates, *bla*_KPC-2_ (61 isolates) was the most frequently identified gene, followed by *bla*_NDM-5_ (18 isolates), *bla*_NDM-1_ (17 isolates), and *bla*_IMP-4_ (1 isolate). Among the 60 PMQR-negative CPKP isolates, *bla*_KPC-2_ (53 isolates) was the most frequently detected gene, followed by *bla*_NDM-5_ (4 isolates), *bla*_NDM-1_ (4 isolates), and *bla*_IMP-30_ (3 isolates). Compared with the PMQR-negative isolates, PMQR-positive isolates harbored fewer *bla*_KPC_ genes but more *bla*_NDM_ genes (*p* < 0.01) (Table 3).

**Table 3:** Antibiotic resistance gene profiles and antimicrobial resistance profiling in PMQR-positive and PMQR-negative isolates

Antimicrobial resistance profiling		CRKPs (*n* = 149)				*P*-values
		PMQR+ (*n* = 89)	%	PMQR− (*n* = 60)	%	
Carbapenem resistance genes	*bla*_NDM_	26	29.2	3	5	< 0.01
	*bla*_KPC_	51	57.3	48	80	< 0.01
	*bla*_IMP_	0	0	3	5	> 0.05
	*bla*_NDM_ + *bla*_KPC_	9	10.1	5	8.3	> 0.05
	*bla*_IMP_ + *bla*_KPC_	1	1.1	0	0	1
ESBL genes	*bla*_CTX-M_	41	46.1	31	51.7	> 0.05
	*bla*_SHV_	5	5.6	5	8.3	> 0.05
	*bla*_CTX-M_ + *bla*_SHV_	26	29.2	21	35	> 0.05
Antimicrobial	Imipenem	88	98.9	58	96.7	> 0.05
	Meropenem	89	100.0	60	100	> 0.05
	Cefoxitin	86	96.6	58	96.7	> 0.05
	Cefotaxime	88	98.9	60	100	> 0.05
	Cefepime	88	98.9	59	98.3	> 0.05
	Ceftazidime	87	97.8	59	98.3	> 0.05
	Aztreonam	79	88.8	58	96.7	> 0.05
	Gentamicin	52	58.4	45	75.0	< 0.05
	Amikacin	35	39.3	40	66.7	< 0.01
	Ceftazidime/ avibactam	28	31.5	9	15.0	< 0.05
	Polymyxin B	2	2.2	3	5.0	> 0.05
	Tigecycline	4	4.5	0	0	> 0.05
	Piperacillin/ tazobactam	80	89.9	54	90.0	> 0.05
	Ciprofloxacin	65	73.0	52	86.7	< 0.05
	Tetracycline	68	76.4	12	20.0	< 0.01
	Minocycline	52	58.4	12	20.0	< 0.01
	Sulfamethoxazole	65	73.0	17	28.3	< 0.01

PMQR+: Represents CRKP isolates with PMQR genes

PMQR−: Represents CRKP isolates without PMQR genes

+: Represents one CRKP isolate harboring two antibiotic-resistance genes simultaneously

*P* < 0.05 was considered statistically significant

*PMQR* plasmid-mediated quinolone resistance, *CRKP* carbapenem-resistant *Klebsiella pneumoniae*, *ESBL* extended-spectrum beta-lactamase

Among the 149 CRKP isolates, PMQR-positive isolates were more sensitive to gentamicin and amikacin but had higher resistance rates to ceftazidime/avibactam, tetracycline, minocycline, and sulfamethoxazole compared with PMQR-negative isolates(*p <* 0.01). PMQR-negative isolates were more resistant to ciprofloxacin due to mutations in QRDRs (*p* < 0.05).

## Discussion

The emergence of CRKP over the past few decades has posed an increasing threat to public health worldwide [2]. FQs have broad-spectrum antimicrobial activities against both Gram-positive and Gram-negative bacteria and have been widely used since the 1980s [29]. In our study, we collected a total of 149 CRKP isolates from clinical patients with BSIs from 11 teaching hospitals across China, and 78.5% (117/149) of the isolates exhibited resistance to ciprofloxacin. Notably, most of the CRKP isolates tested showed high-level resistance to ciprofloxacin and were distributed in eight provinces surveyed in China.

The most prevalent mechanism underlying FQ resistance in *K. pneumoniae* involves mutations in QRDRs. Resistance to FQs is associated with alterations in the GyrA subunit of DNA gyrase and the ParC subunit of DNA topoisomerase IV [30]. Additionally, in 1996, Georgiou et al. reported that some key mutations identified in *gyrA* and *parC* were associated with high-level resistance to ciprofloxacin [31]. In our study, among the 117 ciprofloxacin-resistant isolates, 94.8% had mutations in QRDRs, and all the high levels of resistance (MICs of 16 and 32) were associated with QRDR mutations. Ser80 → IIe in ParC (111/149, 74.5%) was the most common substitution among the 149 CRKP isolates, while Ser 83 → Ile/Phe and Asp87 → Ala/Gly in GyrA were also frequently observed. These GyrA and ParC substitutions observed in this study have already been reported [16]. None of the CRKP isolates harboring mutations in QRDRs were sensitive to ciprofloxacin, suggesting that mutations in QRDRs are the primary cause of FQ resistance. Multiple amino acid substitutions in QRDRs are needed for the acquisition of high-level resistance to FQs [9, 32]. In the present study, isolates possessing double or more amino acid substitutions in QRDRs were highly prevalent, with 105 FQ-resistant CRKP isolates exhibiting at least two mutations within *gyrA* as well as a third mutation in *parC*. The rate of FQs resistance in *K. pneumoniae* has become very high in some parts of Europe, CRKP usually belongs to several genetic lineages, such as the high prevalence of ST11 [33]. Also, ST11 CRKP has become the dominant clone in many China provinces [34, 35]. Similarly, ST11 was the most frequently identified ST among the 117 ciprofloxacin-resistant isolates in our study. Almost all the ST11 isolates had identical mutation patterns in QRDRs and were detected in 8 provinces in China, indicative of wide distribution. This suggests that these isolates, harboring the same FQ resistance gene profiles, may have disseminated vertically by clonal and multiclonal expansion. Similar patterns of the mutation have been reported in clinical Enterobacteriaceae isolated in Warsaw, Poland, but not such large accumulations in clinical CRKP isolates [22].

PMQR determinants (*qnr* and *aac(6′)-Ib-cr* genes) have been found in plasmids and are generally thought to confer only low levels of FQ resistance [36]. A higher rate of PMQR (59.7% 89/149) was detected with CRKP in the current study, which may explain the predominance of PMQR genes among the *K. pneumoniae* isolates [22]. The most frequently detected PMQR gene among all the isolates was *qnrS1*, followed by *aac(6′)-Ib-cr*, *qnrB4*, *qnrB2*, and *qnrB1*, which was not consistent with the results of previous Muggeo et.al results that reported *aac(6′)-Ib-cr* dominance [13]. Nine isolates contained two or more PMQR genes, 1 of which carried four PMQR genes (*aac(6′)-Ib-cr*, *qnr-S1*, *qnrB2, qnrB4*). To the best of our knowledge, this is the first study to report the co-existence of four PMQR genes (*aac(6′)-Ib-cr*, *qnr-S1*, *qnrB2*, *qnrB4*) in CRKP. Among the 31 fluoroquinolone-sensitive CRKP isolates in our study, those positive for PMQR had higher MIC values than their PMQR-negative counterparts, suggesting that PMQR can indeed mediate low levels of drug resistance or increase the MICs of fluoroquinolone-sensitive isolates. Notably, 4 ciprofloxacin-resistant isolates investigated in the present study had no amino acid substitutions in their QRDRs, but all of them had at least one PMQR gene, and all showed considerable resistance levels to FQs (ciprofloxacin MICs of 8 μg/L) by possessing wild-type gyrA and parC. This suggests that PMQR can also mediate drug resistance, although the MIC value was not as high as that seen with some of the CRKP isolates harboring mutations in QRDRs (30 with ciprofloxacin MICs of 16 μg/ml, 14 with ciprofloxacin MICs of 32 μg/ml). Remarkably, in the present study, 1 ciprofloxacin-resistant isolate had no PMQR genes or mutations in QRDRs. We speculate that, in addition to the PMQR genes, other undetected mechanisms may be involved in conferring increased resistance, such as altered permeability or the presence of efflux pump systems.

Nagasaka et al. reported that cephalosporin-resistant *K. pneumoniae* isolates, including those producing ESBL, tend to display resistance to FQs [22]. Additionally, mutations in double-serine residues have often been observed in isolates of the major international STs of ESBL-producing *K. pneumonia* (*gyrA* Ser83 → Phe/Ile; *parC* Ser80 → Ile) [9].

In conclusion, we characterized 149 BSI-associated clinical CRKP isolates from 11 hospitals located in 8 provinces of China and found that PMQR genes and mutations in QRDRs were highly prevalent. Mutations in the QRDRs of *gyrA* and *parC* were key factors underlying FQ resistance in CRKP, while PMQR genes could also increase the level of FQ resistance in the CRKP isolates. Furthermore, the co-existence of PMQR genes and mutations in QRDRs found to be common in this study, led to high levels of FQ resistance. ST11 was the most prevalent ST, while ST11 isolates with identical resistance mechanisms (mutations in QRDRs) were distributed across the eight provinces we investigated, highlighting the need to remain vigilant to prevent its further spread. Additionally, antibiotics, especially quinolones, should be used reasonably in the treatment of clinical CRKP infections.

## Data Availability

The datasets used during the current study are available from the corresponding author upon reasonable request. Most of the data is included in this article. The sequencing data were deposited in GenBank MZ242266-MZ242286.
